# Childhood Emotional Neglect and Cardiovascular Disease: A Narrative Review

**DOI:** 10.3389/fcvm.2022.815508

**Published:** 2022-02-07

**Authors:** Stefan Salzmann, Miriam Salzmann-Djufri, Frank Euteneuer

**Affiliations:** ^1^Division of Clinical Psychology and Psychotherapy, Philipps University of Marburg, Marburg, Germany; ^2^Department of Cardiovascular Surgery, University Hospital Giessen, Giessen, Germany; ^3^Department of Psychology, Clinical Psychology and Psychotherapy, Medical School Berlin, Berlin, Germany

**Keywords:** emotional neglect, cardiovascular disease, mechanism, psychosocial factors, stress

## Abstract

Psychosocial factors predict the incidence and progression of cardiovascular disease (CVD). There is accumulating evidence for the importance of childhood maltreatment for the development and progression of both CVD-related risk factors and CVD. However, past research has predominantly focused on active forms of childhood maltreatment such as emotional abuse, physical abuse, and sexual abuse. At the same time, childhood neglect as a relatively silent form of childhood maltreatment received less attention. Childhood emotional neglect is the most common form of neglect. This narrative review summarizes findings on the association between childhood emotional neglect and CVD and potential underlying mechanisms. These mechanisms may involve biological factors (i.e., elevated inflammation, autonomic dysregulation, dysregulated HPA axis, and altered brain development), psychological variables and mental health (i.e., depression and anxiety), and health behaviors (i.e., eating behavior, smoking, drug use, physical activity) and interpersonal aspects. Evidence suggests that emotional neglect is associated with CVD and CVD risk factors such as obesity, diabetes, inflammation, a dysregulated stress system, altered brain development, depression and other psychological abnormalities (i.e., emotion-regulation difficulties), interpersonal difficulties, and lack of health behaviors. Specific subtypes of childhood maltreatment may be associated with CVD via different mechanisms. This review further encompasses clinical suggestions, identifies research gaps, and has implications for future studies. However, more research with better study designs is desperately needed to identify the exact underlying mechanisms and opportunities for mitigating the negative health consequences of emotional neglect to reduce the prevalence and progression of CVD.

## Introduction

Cardiovascular disease (CVD) is one of the leading causes of disability and mortality worldwide (1, 2). CVD is a chronic disease that develops slowly. It mainly results from the narrowing of the blood vessels providing the heart with oxygenated blood (3, 4). The narrowing of the coronary arteries can result in heart failure (inadequate ejection of blood), arrhythmias (irregular cardiac rhythm), or an acute coronary syndrome (ACS; unstable angina or myocardial infarction). Sudden cardiac death may be a consequence of that. The primary cause for CVD is atherosclerosis, a chronic and progressing inflammatory process (3). A better understanding of predictors for CVD risk and underlying mechanisms may help optimize prevention and treatment for CVD and CVD risk factors. Accumulating evidence indicates that psychosocial factors play a crucial role in CVD incidence and progression (4–6). However, how these factors get “under the skin” is still debated and poorly understood. Childhood maltreatment (CM) is one form of adverse childhood experience (ACE). Emotional neglect as one form of CM has received little attention as a predictor of CVD risk factors and CVD. This review wants to shed light on the associations between emotional neglect and CVD risk.

## Adverse Childhood Experiences, Childhood Maltreatment and The ‘Neglect of Child Neglect'

ACEs are potentially harmful events occurring during childhood and are pretty common (7); estimations range from 52% (8) to 73% (9) of individuals experiencing at least one ACE. As a psychosocial risk factor, ACEs have gained attention in the last years and tend to occur in clusters; this means individuals who experience at least one ACE are likely to experience additional ACEs (7). Consequently, a growing body of work has examined the impact of accumulated adversity rather than the effects of single ACEs (10). In a landmark study, ACEs correlated with several chronic disease outcomes (8). This association was proportional to the number of ACEs. The higher the number of experienced ACEs, the higher an individual's risk for developing a chronic disease later in life; individuals who have experienced ACEs also have an increased risk of developing CVD risk factors (such as obesity, smoking, hypertension, diabetes) and CVD (7, 11, 12). Individuals with at least four or more ACEs have a more than twofold higher risk of CVD onset and an almost twofold higher risk of premature mortality than individuals with none (7). However, different ACEs have frequently been grouped for epidemiological purposes, and past research often did not differentiate between various forms of ACEs (7). ACE is a rather broad term including heterogeneous variables. Therefore, focusing on specific forms of ACEs may offer essential insights, including the opportunity of tailored treatments.

CM as one form of ACEs encompasses abuse (i.e., emotional, physical, or sexual abuse) and neglect (i.e., emotional neglect, physical neglect) under the age of 18. Research has often focused on the role of active forms of CM, such as sexual or physical abuse, while emotional neglect received only a little attention (13, 14). This neglect is astonishing since there is evidence that emotional neglect is at least as damaging as physical or sexual abuse in the long term but has received the least scientific and public attention (14). One explanation for the lack of studies on emotional neglect may be—at least in part—the less visible immediate effects (i.e., no physical injuries or visible signs of abuse) (15). The focus on active forms of CM and the lack of research on (passive) neglect coined the term ‘the neglect of neglect.' (13, 16). Emotional neglect refers to the failure to meet a child's emotional needs; it encompasses experiences such as a child not feeling loved or cared for by family members or does not have the family as a source of support (7, 8). Emotional neglect is highly prevalent: In a meta-analysis in developed countries, the overall estimated prevalence for physical neglect was 16.3%, and 18.4% for emotional neglect (13); the prevalence of emotional neglect is 13.4% in the German population (17). These findings indicate that child neglect is a problem of considerable extent. Experiencing CM is associated with an increased risk for CVD development, hypertension, type 2 diabetes mellitus, and all-cause mortality compared to unexposed individuals (18); however, the role of specific types of CM such as emotional neglect for CVD risk and the underlying mechanisms are still unclear. Since the underlying mechanisms explaining the association between CM subtypes and CVD may be different for different types of CM, it seems even more important to focus on specific CM subtypes' effects. This review focuses on the association between emotional neglect and CVD-related risk factors (such as obesity or diabetes) and CVD and potential underlying mechanisms.

## Emotional Neglect and CVD

### Association Between Emotional Neglect and CVD

Several studies suggest an association between emotional neglect and CVD development (19–21). For instance, a study with over 157.000 individuals from the UK Biobank, assessing all five types of CM (physical abuse, sexual abuse, emotional abuse, emotional neglect, physical neglect) indicated associations for all CM types with a higher risk of CVD (19). This study showed that experiencing emotional neglect increased the CVD risk in men and women in unadjusted and adjusted analyses. The same study also suggested that sex and age may be critical variables when examining CM-CVD associations. When analyses indicated sex differences, associations tended to be stronger in women, and results indicated stronger associations for early-onset CVD (occurrence before the age of 50). In a case-control design study, 75 patients with a first-time CVD diagnosis and 84 healthy participants randomly selected from the general public were compared regarding experienced CM to evaluate whether CM is associated with CVD onset (21). Analyses indicated that the CVD group reported significantly more emotional neglect than healthy controls. However, there is also conflicting evidence indicating no association between emotional neglect and CVD: For instance, a study of 116 patients with Basal Cell Carcinoma showed no association for child neglect with CVD (but for child abuse with CVD) (16). It should be emphasized that this study assessed a very small sample compared to the UK biobank's very large sample. Despite this preliminary evidence on the association between emotional neglect and CVD, it also seems reasonable to assess the association between emotional neglect and subclinical CVD.

### Association Between Emotional Neglect and Subclinical CVD

There is also evidence for the association between emotional neglect and subclinical CVD: 45% of a sample of 295 midlife women reported to have experienced child neglect (or abuse) in their history; emotional neglect (and physical abuse and emotional abuse) was associated with higher subclinical CVD indicated by higher intima-media thickness and carotid plaque than women without CM, while physical neglect was not associated with that difference (22). This finding may be interpreted as that emotional neglect may play a critical but different role in CVD development than other forms of CM, such as physical neglect. However, in another study examining 1.909 adults from the general population without CVD, findings indicated an inconclusive picture for the association between emotional neglect and subclinical CVD (indicated by carotid intima-media thickness and carotid plaque) (23): Emotional neglect was unrelated to both surrogate markers of subclinical CVD.

To date, studies and evidence on the association between emotional neglect and CVD occurrence and progression are limited. Although there is preliminary evidence that emotional neglect is associated with (subclinical) CVD, some studies have not found this association resulting in an inconclusive picture. To gain a clearer picture of the potential association between emotional neglect and CVD, taking a closer look at the potential underlying mechanisms is critical.

## Potential Mechanisms Underlying The Association Between Emotional Neglect and CVD Risk

Previous reviews have suggested that maltreated children show alterations in biological systems related to metabolism and immune system, which may explain the increased risk for developing CVD risk factors (i.e., obesity, type 2 diabetes) and CVD (24). Further evidence suggests that psychological health and behavioral factors may be causally linked to biological processes such as inflammation, which may contribute to and cause CVD (6, 12, 24). However, these reviews did not differentiate between subtypes of childhood maltreatment. Therefore, there remains a need to understand better mechanisms explaining the association between specific CM subtypes, such as emotional neglect and CVD. In the following sections, we focus on potential psychobiological mechanisms specific to emotional neglect and on how these mechanisms may mediate the association between emotional neglect and CVD risk factors and CVD itself.

### Heuristic Model

**Figure 1:**
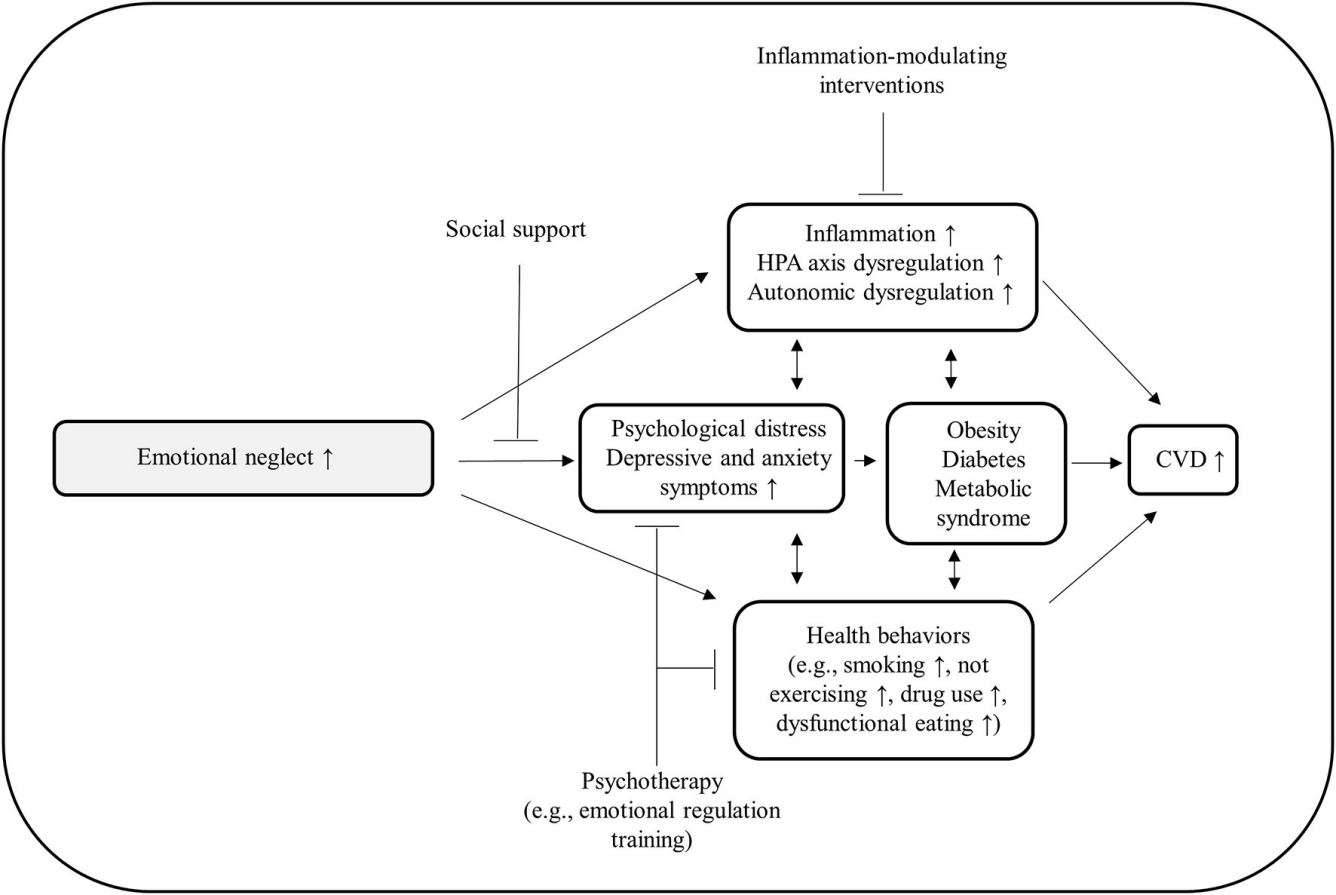
Heuristic model summarizing the associations between emotional neglect and CVD, potential underlying mechanisms, and potential pathways to influence and inhibit pathological processes with psychosocial interventions.

We propose a heuristic model (Figure 1) summarizing the associations between emotional neglect and CVD, potential underlying mechanisms, and potential pathways to influence these mechanisms with psychosocial interventions. In the following sections we will describe the links between emotional neglect, CVD and the potential mechanisms shown in the model in more detail.

### The Importance of the Stress Response for the Association Between Emotional Neglect CVD Outcomes

CM, in general, and emotional neglect, in particular, may lead to chronic activation of the stress response, leading to elevated inflammatory processes, imbalance of the autonomic nervous system, and hypothalamic-pituitary-adrenal axis dysfunction (7, 25). The biological processes of the acute stress response have been well-described (26, 27). According to the Cognitive Activation Theory of Stress (CATS), a stress response occurs whenever a homeostatic imbalance or a threat to homeostasis and the organism's life occurs (28). An “alarm” is set off whenever there is a discrepancy between what should be and what is (discrepancy between a set value and the actual value; i.e., a child's need for love and care and the experienced neglect). The alarm also elicits behaviors to cope with the situation, while the alarm level depends on outcome expectations and responses available for coping (28). The stress response can be seen as an essential response aiming to restore homeostasis (i.e., receiving the emotional support a child needs). The unpleasantness of the stress response is no health threat itself. This response is adaptive, aims to restore homeostasis, and inactivation returns the systems to baseline levels which usually happens after the danger (or discrepancy) is gone (i.e., a child cries and receives support/care); however, if the inactivation is inefficient or the activation happens too often (i.e., due to an ongoing discrepancy between emotional needs and perceived emotional care and support)—over time—this can result in pathophysiological processes leading to allostatic load—which can be seen as a biological multi-system dysregulation—and chronic stress (26, 29).

Chronic stress and allostatic load as a consequence of emotional neglect may then lead to subsequent development of risk factors such as diabetes, hypertension, dysfunctional health behaviors such as smoking, physical inactivity, heavy alcohol consumption, inadequate sleep hygiene, and non-adherence, which in turn result in the onset of CVD including increased morbidity and increased mortality rates (5, 7, 30). For instance, a study assessing the association between prolonged parent-child separation and allostatic load as a measure of the physiological wear-and-tear indicated that the allostatic load [measured by 11 biomarkers such as inflammatory parameters (CRP), indicators of the metabolic system (body mass index, high/low-density lipoprotein, total cholesterol, triglycerides, fasting glucose, glycated hemoglobin, insulin) and blood pressure] was higher in children separated from both parents during childhood or persistently since birth compared to those not having experienced parental separation (31). Predictors for higher allostatic load levels were (after adjustment for demographic covariates) body mass index, parent frequency of communication, and parental warmth, as well as persistent separation or separation from both parents. Research indicates that early childhood is a particularly vulnerable period of life to the effects of stress since this life state is crucial for the development of behavioral, metabolic, brain, and immune abnormalities (25).

## Biological Factors

### Elevated Inflammation

Elevated levels of circulating pro-inflammatory parameters such as Tumor Necrosis Factor (TNF)- α, Interleukin (IL)-1β, IL-6 IL-8, or CRP indicate systemic inflammation. Inflammation has reliably been associated with CVD incidence and progression and lower baseline inflammatory levels, for instance, predict better long-term CVD outcomes (3, 32).

A meta-analysis assessing the association between ACEs (including CM and subtypes of CM) and inflammatory parameters showed that individuals who experienced ACEs had elevated baseline CRP, IL-6, and TNF-α levels (33). Interestingly, subgroup analyses for different ACEs indicated that different trauma types influenced single inflammatory parameters differentially: CRP seemed to be increased primarily in individuals with parental absence (which can be seen as one form of emotional neglect) during early development. At the same time, physical and sexual abuse were associated with increased TNF-α and IL-6 levels, but not CRP. This meta-analysis thus argues for the notion that CM leads to an elevated pro-inflammatory state in adulthood and that the specific inflammatory profile may depend on the type of trauma experienced. A systematic review also concluded an association between CM in general and elevated CRP levels in prospective studies (34). However, this review also indicated that the included retrospective studies' findings and other biomarkers were conflicting. The review further showed that the included studies varied substantially regarding the assessed outcomes (i.e., only some studies assessed CM subgroups, whereas others focused on the overall severity of child maltreatment). One of the few included studies on emotional neglect indicated that retrospectively reported emotional neglect during childhood was associated with elevated inflammatory levels (indicated by CRP) (35). While other forms of CM (such as emotional and physical abuse and physical neglect) were associated with elevated CRP levels, mediated by body mass index (BMI) levels, the association between emotional neglect and CRP levels was not mediated by the BMI. After adjusting for other ACEs and confounding variables, emotional neglect was the only ACE subtype showing an association with glycoprotein acetyls (a marker of chronic and cumulative inflammation) in a large birth cohort study assessing two generations (36). These findings suggest that the association between emotional neglect and inflammatory variables may be mediated via a different pathway than other CM subtypes.

Although long-lasting elevations in CRP levels due to emotional neglect may not be comparable with CRP elevations due to acute inflammation, these slightly higher levels—compared to individuals without experiencing emotional neglect—may still have clinically relevant effects. CRP predicts hypertension (37), CVD, and future cardiovascular events (38, 39) and has been divided into subgroups. The following three categories have been developed in epidemiologic studies examining CRP levels to predict future cardiovascular events: low risk, <1.0 μg/ml; average risk, 1.0–3.0 μg/ml; and high risk, >3.0 μg/ ml (40). Given that CM such as emotional neglect lead to elevated CRP levels in affected individuals [individuals with a history of childhood trauma show an average CRP increase of 0.84 μg/ml (33) compared to healthy individuals], experiencing emotional neglect in the past may put these individuals in the next higher CRP risk group for future CVD events compared to individuals who have not experienced emotional neglect.

To conclude, experiencing emotional neglect during childhood seems to be associated with increased CRP levels; however, only a few studies specifically looked at emotional neglect and the associations with inflammatory parameters.

### Autonomic Dysregulation

Dysregulation of the autonomic nervous system (ANS), including the sympathetic and parasympathetic nervous system, interacts with the immune system's pro-inflammatory processes (41–43). Therefore, the ANS may play a critical role in physiological processes in individuals who have experienced emotional neglect. However, studies explicitly assessing the association between emotional neglect and the ANS remain scarce. One study indicated that childhood neglect might lead to sleep inefficiency via a dysregulation in arousal (44). In contrast, in a large multi-ethnic cohort study examining an urban population, CM (including emotional neglect) was associated with a higher CVD rate and reduced baroreflex sensitivity and heart rate variability (45). However, while the association between CM and CVD remained stable, the association between CM and autonomic regulation vanished when adjusting for demographic, health, and psychological factors. The finding that the number of reported CM types was not associated with autonomic regulation over and above the effect of demographic, health, and psychological factors is essential; however, other studies are necessary to draw sound conclusions regarding the role of the ANS in emotional neglect.

There is further preliminary evidence that CM may alter the body's ability to respond to stress in adulthood adaptively and that sex may be an important moderating variable (46): In an experimental study, 453 healthy young adults underwent two identical stress inductions (4-minute acute psychological stress task) and reported CM experienced. Women, but not men, with higher self-reported CM showed less habituation of heart rate and diastolic blood pressure across the stress tasks. This may be an important finding since it has been suggested that failing to habituate to repeated stressors is a risk factor for CVD development and progression (47, 48). However, it should be mentioned that the findings in this experiment were not specific to emotional neglect. More evidence seems to be available when taking a look at other body systems involved in stress regulation.

### Dysregulated HPA Axis

Emotional neglect has been shown to have long-term effects on neurobiological functioning: children experiencing the absence of responsive caregiving (which can be seen as one form of emotional neglect) show a dysregulated HPA axis activity indicated by diminished diurnal cortisol production, particularly low morning cortisol (49). However, several interventions have been shown to be able to normalize cortisol production in maltreated children in terms of approaching more normal cortisol patterns, which are associated with enhanced behavioral and emotional outcomes (49, 50). There is also evidence from animal studies indicating that early life stress such as distorted caregiving and maternal separation has a negative impact on behavioral factors and the neuroendocrine stress response system in rodents and non-human primates (51). Further evidence from non-human primate studies showed that early life stress in terms of impaired maternal care was causally associated with an increased risk of developing obesity and insulin resistance (52, 53). It has been suggested that experiencing CM may lead to a “thrifty” phenotype characterized by increased energy intake and storage combined with decreased energy expenditure (54). An altered HPA axis activity may play a crucial part in the association between emotional neglect and CVD risk due to its essential regulatory role for various biological processes.

There is only scarce evidence on the role of emotional neglect for the acute response of the HPA axis due to acute social stress: In a systematic review assessing the association between salivary responses as a response to acute stress due to the Trier Social Stress Test (TSST) and ACEs, only one (out of 12) study indicated a negative association between emotional neglect and cortisol responses (55). However, only three out of the 12 included studies assessed associations between CM subscales and cortisol levels. The review concluded that the association between CM in general and specifically for emotional neglect and cortisol responses to a stress induction as well as the association between CM and cortisol responses to acute psychosocial stress remain inconclusive.

In one study, childhood neglect was associated with a more robust psychological stress and physiological (higher ACTH levels) response in patients with type 2 diabetes (which was not present in healthy individuals) (56). These results may indicate a link between childhood neglect and a dysregulated physiological stress response in type 2 diabetes patients. However, there is a substantial need for future longitudinal studies assessing the association between emotional neglect and cortisol responses.

### Altered Brain Function and Development

Child neglect is associated with delayed cognitive and emotional development (57). Experiencing CM influences certain brain areas and their functions in the developing brain; especially areas such as the hippocampus (involved in learning and memory), amygdala (relevant for anxiety and fear), and the prefrontal cortex (emotion regulation and cognition) seem to be negatively influenced by childhood maltreatment, particularly by emotional neglect (7, 25). Growing evidence suggests that neglect may substantially affect the human brain, such as myelination and white matter integrity. Emotional neglect may cause abnormal development of an individuals' bonding system, may increase aggressiveness and preference for addictive substances. At the same time, it may decrease social competence and the ability to cope with stress experience rewarding interpersonal relationships (58). Converging molecular pathways may explain how CM increases the susceptibility to mental disorders in adulthood (59). Emotional neglect has been found to be associated with blunted development of reward-related ventral striatum (VS) activity in 106 participants (ages 11–15) between the first and the second assessment two years later (60). In addition to that, reward-related vs. activity correlated to depressive symptoms and partially mediated the association between emotional neglect and depressive symptoms. Low reward sensitivity may contribute to satisfying this reward deficit with high-caloric and unhealthy food in children having experienced emotional neglect (24). A dysfunctional development of the prefrontal cortex in maltreated children may decrease a child's executive function, including impaired inhibitory control regarding food intake (61), resulting in increased energy intake.

CM subtypes have also been found to have differential effects in neural threat reactivity (62). Findings like these provide important insights and may indicate the effects of emotional neglect on the brain leading to mental health adversities. One study assessed a sample of 48 Black mother-neonate dyads with self-report measures, and fMRI scans at one month postpartum of the newborns during natural sleep (63). This study indicated that greater maternal exposure to emotional neglect during childhood was associated with more robust functional connectivity of two different frontoamygdala circuits in neonates. These findings are interesting since connectivity between these regions has been found to increase in the context of fear learning (64) and in response to acute stress (65). Even more critical, such findings show that experiencing early adversity in emotional neglect may also be transmitted across generations.

## Psychological Variables and Mental Health

### Depression

Research has demonstrated that psychological factors and mental health can substantially affect cardiovascular health (6). There is further strong evidence that experiencing CM in general increases vulnerability for mental health problems (7, 66). Compared to other forms of ACEs and CMs, meta-analyses have shown that emotional neglect and abuse have the strongest associations with depression in adults (67–69). These findings are essential since solid evidence shows that depression is a highly relevant risk factor for CVD (70, 71). Interestingly, emotional neglect seemed to be a more important predictor of adverse mental health in adulthood than physical neglect and was associated with depression, anxiety, and stress (72).

Further evidence for the role of mental health and protective factors stems from extensive longitudinal studies: In a longitudinal birth cohort study assessing emotional neglect across seven assessments from 8 to 17.5 years, peer social support at age 15, and depressive symptoms at age 18 in 3.265 individuals from the Avon Longitudinal Study of Parents and Children (ALSPAC) in the UK, higher levels of emotional neglect were associated with higher depressive symptoms, while higher peer social support was associated with lower depressive symptoms (73). However, peer support did not interact with depressive symptoms. The results of this study provide evidence for emotional neglect as a risk factor for depression; however, it further suggests that social support at age 15 is a protective factor. Interventions that foster strong peer social support during adolescence may help reduce the risk for depression later in life, even for individuals experiencing emotional neglect during childhood, thus decreasing the associated CVD risk.

In another longitudinal study, 673 adolescents completed self-reports for CM and depressed mood as well as anhedonia over six years in a row (74). Both emotional abuse and emotional neglect predicted levels of depressed mood over time, while only emotional neglect predicted levels and trajectories of anhedonia. Interestingly, physical and sexual abuse was not associated with depressive symptoms when adjusting analyses for emotional abuse and neglect. These results seem to align with recent findings that more silent types of maltreatment uniquely predict depression. This has significant implications since abuse and neglect may cause distinct risk profiles for psychological distress, which may require different treatment approaches. Emotional neglect has further predicted increased depressive symptoms, while (decreased) emotional clarity mediated this association (75). Interestingly, emotional abuse did not predict emotional clarity. Results like these suggest that specifically emotional neglect (but not emotional abuse) may decrease an individual's ability to identify one's own emotions, which may increase depressive symptoms during adolescence. Interventions aiming to increase one's emotional clarity may buffer the effects of emotional neglect on depressive symptoms and may thus reduce the risk for developing CVD.

Depressed adults show elevated inflammatory parameters; however, there remains substantial heterogeneity regarding inflammatory levels in depressed individuals (76–78). One study examined the possible role of individual differences in exposure to CM in contributing to cytokine level variability in depressed adolescents (79): The study assessed 52 depressed and 20 healthy adolescents and measured CM and cytokine levels IL-6 and TNF-α cross-sectionally and longitudinally (for a subgroup of depressed individuals). The authors found a positive association between higher CM and TNF-α in depressed adolescents but no association between CM and change in cytokine levels longitudinally. The CM subtypes emotional neglect, physical neglect, and emotional abuse were also associated with increased TNF-α levels; however, after adjustment for multiple comparisons, these associations were not statistically significant anymore. The finding of elevated TNF-α levels (although not significant) with emotional neglect is interesting since experiments in rodent models have shown that maternal separation is associated with increased TNF-α levels in prefrontal and hippocampal brain regions in adult life (80). However, it is unclear why other studies found that emotional neglect during childhood seems to be associated with increased CRP levels (33) but not with elevated levels of TNF-α. Taking a closer look at different CM subgroups may help explain the heterogeneity of cytokine levels in depressed adolescents offering the opportunity to guide more effective individualized treatments for individuals with depression; however, there remains substantial uncertainty, and future studies are necessary.

### Anxiety

Emotional neglect has also been found to be associated with anxiety symptoms such as generalized anxiety disorder symptoms (81) and post-traumatic stress disorder (PTSD) scores (82). These findings are relevant since anxiety (disorders) have also been shown to be predictors for CVD outcomes (30, 83). In a longitudinal, multi-wave study with 580 adolescents, the ‘neglect' group showed significantly elevated depression, PTSD, illicit substance abuse, and cigarette use compared to the “no trauma” group (84). Of note, CM has also been found to predict personality disorders, with emotional abuse and emotional neglect showing the highest risk for developing borderline personality disorders later in life (85).

The importance of anxiety and depression as mediating variables—which may explain the association between emotional neglect and CVD—also stems from extensive population studies: Data from over 40,500 men and over 59,500 women from the UK Biobank were used to estimate the indirect effect of CM on incident CVD (86). Results indicated that all forms of CM were associated with an increased CVD risk. Anxiety/depression, smoking, BMI, and inflammation-mediated 26–90 % of the association between CM and CVD, while the extent of the mediation varied by type of maltreatment and sex. For emotional neglect, emotional abuse, and sexual abuse, anxiety/depression mediated the most significant proportion, especially in women. In men, BMI was the most critical mediator explaining the association between physical abuse and physical neglect with CVD.

## Health Behaviors and Interpersonal Aspects

### Eating Behavior

Health behaviors have been proposed as another essential mechanism explaining the association between CM and CVD (7). In a population-based, longitudinal study with over 1.600 individuals, having experienced CM was associated with a more than 60% greater risk for chronic dieting and overeating, with additional associations found for binge eating, weight and shape concerns, and unhealthy weight control behaviors (87). Interestingly, in this study, ACEs in general, but emotional neglect in particular and to the greatest extent, was a significant risk factor for problematic eating behaviors in adult men and women. These findings indicate that especially emotionally maltreated individuals have an increased risk for obesity due to altered eating behavior. Since maltreated children indicate a chronic overactivation of the HPA axis which can be associated with physical and mental anxiety symptoms (88), and supposed that high-caloric food can decrease the HPA axis' activity and reduce anxiety symptoms, individuals may use high-caloric food as “self-medication” (24). This behavior may lead to increased caloric intake, promoting obesity. CM has been shown to be associated with adult obesity: In an epidemiological study including 2.936 Germans, overall CM was associated with a higher waist-to-height ratio (WHtR) for men and women (89); however, this association was not significant anymore in women after adding sociodemographic covariates but remained significant in men. Interestingly, emotional neglect and abuse showed a more substantial impact on WHtR in women than in men, while physical neglect and abuse seemed to be stronger predictors in men.

### Smoking and Drug Use

Emotional neglect has also been associated with significantly higher smoking compared to a “no trauma” group in longitudinal analyses (84). Of note, individuals reporting childhood abuse also indicated higher smoking levels than the “no trauma” group, while there was no significant difference to those with neglect. In another study, emotional neglect (while controlling for other types of CM) was associated with riskier behaviors (such as lower age at first consensual intercourse and not using seatbelts) (90). Emotional neglect (as well as other types of CM) is further associated with higher suicidal ideation (91) and higher rates of suicide attempts (92), drug use such as opioid use disorder (93), and risky sexual behavior (94) as well as disturbed sleep (44).

### Interpersonal Aspects

There is evidence that maltreated children are more likely to show lower levels of prosocial behavior and higher levels of disruptive, aggressive and withdrawn behavior, which was longitudinally associated with an altered HPA-axis activity (lower morning, but higher afternoon cortisol levels) (95). Due to their vulnerability for difficulties in social relationships, emotionally maltreated children seem to be at risk for altered cortisol regulation. Experiencing stressful situations due to increased problems to handle social situations adequately may exhaust the body's stress systems in the long run. Interventions which may help to improve a child's social skills may be able to reduce a child's daily stress and may thus normalize HPA axis activity. Young children with low morning cortisol levels may show more normal cortisol patterns with improved care (50): Studies of orphanage-reared children show low morning cortisol levels, while those children adopted indicated to approach the levels of family-reared children. However, whether this applies to all CM subtypes, such as emotional neglect, is not known. Emotional neglect may lead to problems in social relationships due to problems in emotion regulation (96): the effects of emotional neglect on social relationship problems have been shown to be mediated by antecedent-focused difficulties in emotion regulation encompassing awareness and understanding of one's own emotions. In combination with the finding that (decreased) emotional clarity is an essential mediator between emotional neglect and depressive symptoms, difficulties in emotion recognition and regulation seem to be key mechanisms for depression and interpersonal relationships. It has been hypothesized that difficulties in emotional clarity may stem from lacking emotional models during childhood (96). A history of emotional neglect has further been associated with loneliness, while this association was mediated by rejection sensitivity in patients with persistent depressive disorder and borderline personality disorder (97). Plasma oxytocin levels and attachment representation have been found to be mediating the association between experiencing emotional neglect during childhood and social dysfunction indicated by fear and avoidance of social situations in adulthood in a population-based cohort (98). Problems in social relationships may contribute to social isolation and loneliness, which have both been shown to be independent risk factors for CVD and mortality (99, 100).

### Physical Activity

Having experienced emotional neglect may also be associated with reduced physical activity due to an increased risk for depression and sickness behavior induced by increased inflammatory levels. Depression and inflammation have both been found to be associated with physical inactivity (101, 102). Pro-inflammatory biomarkers such as IL-6 or IL-8 change brain functions and can induce sickness behavior indicated by depressive-like behavior such as impaired mood and reduced willingness for activities such as physical activity (103–105). A lack of physical activity in emotionally neglected children leads to a decreased energy expenditure, which may—in combination with an increased caloric intake (as described above)—contribute to obesity.

## Other Psychological Factors

Important psychological mechanisms which may mediate or buffer the deleterious effects of emotional neglect on physical and mental health outcomes may encompass perceived personal control, self-esteem, self-compassion, self-, and emotion-regulatory and adequate coping mechanisms. For instance, it has been shown that the lack of parental support (which can be seen as an indicator of emotional neglect) during childhood leads to lower personal control beliefs, lower self-esteem, and poor social relationships (106). In the same study, individuals with high perceived personal control and self-esteem had fewer depressive symptoms.

A meta-analysis indicated that emotional neglect was negatively correlated to self-compassion (107). This finding is essential since self-compassion is considered an essential protective factor after a child has experienced neglect. It may be one mechanism that underlies resilience and effective emotion regulation (108). In a non-clinical community sample of young adults, poor psychosocial outcomes such as heightened stress perception, decreased social support, and reduced emotional well-being were associated with a CM total score, but also with emotional neglect and physical neglect, and emotional abuse (109).

It is essential to bear in mind that all mechanisms mentioned so far probably influence each other rather than working independently. In addition, it may be essential to consider other psychosocial risk factors such as socioeconomic status since the risk for emotional neglect is associated with low socioeconomic status in individual studies (13). Besides a better understanding of how emotional neglect may cause CVD, it is also essential to better understand which exact mechanisms buffer or hinder these processes.

## Discussion

In summary, there is good evidence that emotional neglect increases the risk for developing depressive or anxiety symptoms, which are critical predictors for CVD risk factors and CVD themselves (Figure 1). There is further evidence that emotional neglect increases the risk for unhealthy behaviors such as smoking, increased caloric intake, drug use, physical inactivity and interpersonal difficulties. Preliminary evidence indicates that emotional neglect is associated with increased inflammatory levels (i.e., CRP) and an altered HPA axis activity. Weak evidence indicates that emotional neglect may be associated with autonomic dysregulation. These factors can pose risk factors for developing CVD risk factors such as diabetes, obesity, hypertension, or metabolic syndrome. In turn, these factors may themselves contribute to CVD development; however, it is unclear whether the variables mentioned are adding directly or indirectly (via other variables) to CVD risk.

Protective factors or opportunities to buffer the deleterious effects of emotional neglect on CVD outcomes may encompass social support, psychotherapy, emotion regulation training, and self-compassion. At the same time, anti-inflammatory interventions may decrease an individual's chronic inflammatory state.

### Clinical Suggestions

Given the high prevalence, relevance, and costs of CM (105), primary prevention of CM in general and emotional neglect, in particular, should be prioritized. Politics and society should aim to create contexts and conditions that reduce the occurrence of emotional neglect in the first place and offer support for those suffering from emotional neglect themselves or who are at risk of neglecting their children. Emotional neglect's severe and long-lasting consequences may argue for efforts and increased investments in early identification and preventive and therapeutic strategies. More and more evidence linking emotional neglect and CVD outcomes accumulate and stress the need for more psychological and psychotherapeutic efforts in patients suffering from CVD due to the high relevance of mental health and health-related behaviors for CVD development and progression (6). Especially the evidence on the critical role of depression as a frequent co-morbidity in CVD patients and the role of emotional neglect in the development of depressive symptoms suggests that an interdisciplinary approach and more involvement of mental health professionals in somatic areas is critical. An (earlier) referral to psychotherapy for vulnerable individuals may be helpful since psychotherapy such as mindfulness-based, cognitive behavior therapy, and physical exercise may mitigate the effects of CM on cardiometabolic outcomes (7). Fostering social support may help reduce the risk for depression in individuals at risk due to experienced emotional neglect during childhood (73). Adults and adolescents experiencing problems in interpersonal situations may benefit from the training of emotion regulation skills (including training of understanding and assessing one's own emotions) or social competence training (i.e., to train how to solve interpersonal conflicts or how to foster positive relationships). A first step to improve clinical practice could be to raise awareness in clinicians for the importance of emotional neglect and to screen for CM and other ACEs in CVD patients. Providers such as the general practitioner or the cardiologist could ask the patient to fill in brief questionnaires such as the short form of the Childhood Trauma Questionnaire as a screening measure (110). Knowing who has suffered from emotional neglect during childhood could help offer additional support for those in need. A better understanding of the exact mechanism relating emotional neglect to CVD would offer directions for the most effective interventions: for instance, if obesity would be the central mediator between emotional neglect, inflammation, and CVD, effective interventions would probably encompass exercise, reducing weight, and dietary change. Suppose there is strong evidence for a more direct role for inflammation. In that case, an anti-inflammatory medication may be an effective intervention to reduce the CVD risk in individuals with emotional neglect (34).

### Future Research

Recent reviews on ACE and CM have expressed the need for more robust study designs: Childhood adversities are often assessed retrospectively, which may lead to distortion in its estimation since the agreement between retrospective, and prospective assessments of ACEs is poor (11). In addition, most studies so far are observational, while only a few of the studies used prospective data collection. However, even prospective studies have to be interpreted with caution since pharmacological control of CVD risk factors (i.e., blood pressure, cholesterol, diabetes) have been shown to cause reduced CVD event rates, although the indication for these pharmacological treatments may be a risk factor for worse CVD outcomes (111, 112). Available studies show significant variations in both theoretical and methodologically approaches (continuous vs. dimensional coding of CM, retrospective vs. prospective assessment, compound score vs. individual CM subtypes such as emotional neglect, adjusting for different covariates and other ACEs experienced vs. no adjustment in the analyses, amount or frequency vs. severity of CM). Therefore, aggregating evidence on the best methods and developing guidelines that future studies can follow may help compare results between different studies. Different approaches to treating several CM as a cumulative burden vs. grouping them in clusters are different approaches that may be necessary but will answer different research questions (113). It seems that emotional neglect has more in common with emotional abuse than, for instance, sexual abuse; however, more studies on that distinction are warranted. There may also be subgroups especially vulnerable to emotional neglect: For instance, individuals with adult congenital heart disease (ACHD) reported significantly higher rates of emotional neglect and emotional abuse and sexual abuse and lower rates of physical neglect when compared to the general German population (114).

Since preliminary evidence suggests that different types of CM are associated with different inflammatory parameters and distinct psychological processes [i.e., CRP and emotional clarity may play a more crucial role than in other forms of maltreatment (33, 96)], future research should examine the exact mechanisms. These efforts should encompass examining which brain regions may be activated by what kind of CM and which processes and stress responses may be the differential consequences. A better understanding of the biological and psychological correlations of different CM subtypes will help identify the exact mechanisms involved and would thus offer specific treatment targets. A better understanding of psychological protective factors and how to boost resilience are also important future research questions to develop powerful interventions able to mitigate the deleterious effects of emotional neglect. In particular, it would be exciting to assess the psychological effects of psychological interventions and the interventions' biological effects since psychosocial interventions can enhance an individual's immune system (115).

## Conclusion

In summary, there is accumulating evidence for the importance of experiencing emotional neglect during childhood and the associated risk for CVD. Emotional neglect seems to increase the risk of developing and progressing CVD-related risk factors and CVD itself. Emotional neglect is predominantly associated with increased depressive and anxiety symptoms during adolescence and adulthood, increased inflammatory levels, altered HPA axis function, and altered brain development. Future research should aim to identify opportunities for mitigating the negative health consequences of emotional neglect to reduce the prevalence and progression of CVD, such as fostering peer social support. Current evidence supports the notion that emotional neglect may unpack its deleterious effects via different psychobiological mechanisms than other forms of CM. Given the high prevalence and the neglect of this critical adversity, more studies should aim to elucidate the associations with health outcomes and to assess underlying mechanisms to develop optimal treatment strategies. Focusing more on emotional neglect in CVD treatment may not only help reduce CVD burden but treat the whole person rather than CVD alone.

## Author Contributions

All authors contributed significantly to the conception of this article, the manuscript draft, the critical revision of contents, provided approval for publication, and agreed to be accountable for all aspects of the work.

## Conflict of Interest

The authors declare that the research was conducted in the absence of any commercial or financial relationships that could be construed as a potential conflict of interest.

## Publisher's Note

All claims expressed in this article are solely those of the authors and do not necessarily represent those of their affiliated organizations, or those of the publisher, the editors and the reviewers. Any product that may be evaluated in this article, or claim that may be made by its manufacturer, is not guaranteed or endorsed by the publisher.
